# Co-creation in a digital health living lab: A case study

**DOI:** 10.3389/fpubh.2022.892930

**Published:** 2023-01-17

**Authors:** Theofanis Fotis, Kitty Kioskli, Anand Sundaralingam, Amer Fasihi, Haralambos Mouratidis

**Affiliations:** ^1^School of Sport and Health Sciences, University of Brighton, Brighton, United Kingdom; ^2^School of Computer Science and Electronic Engineering, Institute of Analytics and Data Science (IADS), University of Essex, Essex, United Kingdom; ^3^Trustilio B.V., Amsterdam, Netherlands; ^4^Oxford Respiratory Trials Unit, Nuffield Department of Medicine, University of Oxford, Oxford, United Kingdom; ^5^Kraydel Ltd., Belfast, United Kingdom

**Keywords:** living lab (LL), living lab approach, living lab design, digital health, innovation, co-production, co-creation

## Abstract

Co-creation in healthcare, especially in developing digital health solutions, has been widely identified as a fundamental principle for person-centered technologies that could accelerate the adaptation of innovation. A Digital Health Living Lab based on community offers a sustainable and real-life environment to ideate, develop, and evaluate digital health solutions addressing the needs of multiple stakeholders. This article presents the experience of the School of Sport and Health Sciences at the University of Brighton in establishing a Digital Health Living Lab. In addition, we share a proposed step-by-step approach to establishing such a living lab in the community, supplemented by a case study of product development.

## Introduction

Innovation in digital services and products is mostly dependent on enhancing knowledge on a national and international scale, targeting to foster an ecosystem of complementary evidence (1). Therefore, it has become broadly accepted that the innovation process would be leveraged by including external stakeholders from the early stages to create a competitive advantage. Meanwhile, users are also encouraged to be involved. However, the feasibility of such involvement is debated in the literature (2). The living labs are user-centric innovation tools that have become very prominent in recent years (3) to fulfill this vision.

Over the last two decades, numerous initiatives, organizations, and institutes have sprung up worldwide as “living labs.” Meanwhile, regional and national governments, as well as international bodies (i.e., European Commission) have cautiously supported the concept of “living labs” and included it in their work programs (4–6). Overall, the phenomenon of a living lab mainly refers to and supports the involvement of multiple stakeholders for the (co-) creation, application, and evaluation of innovation services or products within a real-life setting (7, 8). Currently, there are numerous living labs worldwide, but a higher concentration is observed in Europe (9).

There is no standard definition of the concept of the living lab. However, according to the European Network of Living Labs (ENoLL) (10), these are defined as “user-centered, open innovation ecosystems based on systematic user co-creation approach, integrating research and innovation processes in real life communities and settings” (10). ENoLL's definition aligns with many definitions found in the literature. For example, the article by Leminen et al. (11) defines the living labs similarly as “physical regions or virtual realities in which stakeholders form public–private–people partnerships of firms, public agencies, universities, institutes, and users of products or services, all collaborating for creation, prototyping, validating, and testing of new technologies, services, products, and systems in real-life contexts.”

The living lab phenomenon embraces different contexts, for instance: the development of innovation activities driven by citizens aiming to improve everyday life, testing of technology-driven human-centric products from pharmaceutical companies, targeting to provide affordable and easy-to-use products to patients, or development of activities from NGOs, citizens, or other actors in developed societies (12). These differentiated actions may be initiated by various stakeholders (i.e., providers, users, and enablers), which impact the duration, focus, and outcomes of the innovation actions (11). Overall, the living labs offer a safe space for development, testing, and validation with co-creation in all stages, from conceptualization to commercialization (13). They are often characterized as testbeds for innovative solutions, systems, and products by providing a platform for collaboration (14, 15).

A living lab involves the following four main pillars:

- Co-creation: Co-design by users and producers.
- Exploration: Discovering emerging usages, behaviors, and market opportunities.
- Experimentation: Implementing live scenarios within communities of users.
- Evaluation: Assessment of concepts, products, and services according to socio-ergonomic, socio-cognitive, and socio-economic criteria.

It is worth noting that a core element of any living lab is sustainability, and there is a plethora of studies that have addressed this (16), but their perspectives differ. For example, some studies examine development and innovation activities that target to improve, in a sustainable way, the everyday life of citizens (12). At the same time, other studies explore transition labs that aim to accomplish change in sustainable development (17) or analyze the connection between living labs and sustainable innovation (13). Others investigate the role of processes, design, and practice in environmental transformation (18). Moreover, studies have also focused on sustainable development in smart city actions (19) and in entrepreneurship and urban development (20).

The existing literature gives a fruitful basis for understanding the potential and usefulness of living labs. This is due to their conceptualization and theorization (21), which further investigates the processes and methods followed (22), while also recording results from empirical studies (23–25). More specifically, Følstad (26) wrote the first review study, including 32 articles to establish theoretical foundations, methods, perspectives, and processes of a living lab. Later, Franz (27) developed an understanding of the phenomenon, which was socially centered. Schuurman et al. (5) reviewed 45 studies and concluded that practice and research in the living labs were still in the infant stages. Research in the existing literature from Leminen and Westerlund (28) established eight main research streams at the time. After reviewing 195 studies, Leminen et al. (6) aimed to understand the need around the emergence of the living labs movement. McLoughlin et al. (29) conducted a bibliometric analysis of 169 studies, while a more recent study by Westerlund et al. (30) performed topic modeling for 86 articles on the topic.

More recently, Hossain et al. (31) conducted a systematic review of 114 studies regarding living labs to gain an understanding of the main facets discussed in the developing literature. Notably, the study investigated the origin of a living lab and its key characteristics and paradigms, including contexts, stakeholder roles, main outcomes, challenges, and sustainability. It is notable that the literature in the living lab context has increased vastly since 2015, showing the urgency and advantages of the phenomenon. Scholarly studies discuss the living labs as infrastructures that could be utilized as novel tools for research opportunities to tackle needs and challenges in society (32). However, it is evident that the literature on living labs in the context of digital health and the linkage to innovation is still rather fragmented (33).

Traditional models of healthcare are experiencing significant pressure in the context of overwhelming strain on the existing systems due to the high demand for services from one side and limited funding from the other (34). As a result, an important opportunity for innovation in digital health has arisen. However, the market is currently leading in innovation in this space, but there is significant risk in bringing healthcare products and/or services that are not evidence-based to be consumed directly by the masses. “Disruption” is often proclaimed as the mark of any worthy innovation. However, this adopts a rather irresponsible view. In many cases, it is this irresponsible view that results in tensions between technologists and healthcare professionals (35).

The difficult issue of evidence-based digital health often rears its head, and it is a challenge not just for the technologists who are operating in a rather unfamiliar space but for the academics too. The bridge between academia and industry that aims to create links between the two sides is on the rise, but it is still not fully robust. Although the relationship seems simplistic, they function in parallel. Filling the gap through a fully developed collaboration in research between the two can boost the economy and growth by preparing a much-needed workforce with the industry's required skills and products that are developed and evaluated through evidence-based academic methodologies (36). Given that digital health interventions are often sitting at the intersection of biomedical, behavioral, and computer science, not to mention the design and user experience components, technologists feel that the classical evaluation models do not do them justice. Enhancement by collaboration from different disciplines is urgently needed (37).

The benefits to be gained from the participation of end-user groups, local health and government organizations, voluntary sector organizations, technologists, and Academic Health Science Networks (AHSNs) cannot be overstated. As outlined in Greenhalgh et al. systematic review of the challenges related to adapting new technologies, there are many obstacles to sustaining technological change, many of them resulting from the complex adaptive systems that provide healthcare (38). From this review, the Non-adoption, Abandonment, Scale-up, Spread, and Sustainability (NASSS) framework was derived as a tool to explore and identify sustainable adoption and applicability of technological innovations in healthcare and social care. The living lab model provides an opportunity to explore this complexity and shed light on the wider system which is targeting to embed the change with evidence-based models. Genuine stakeholder participation can be used as a tool for the ideation, development, and evaluation of digital health solutions toward optimizing the conditions within the system and refining the technology to match the requirements of the system. As a result, it will create a sustained and scalable technological change with rewards that are realized and provide benefits to all involved actors.

In this study, we present the setting up of a community-based Digital Health Living Lab (DHLL).

### Living lab environment

The lab was the culmination of a national scheme, the Leading Places program, that had brought together the University of Brighton with Brighton and Hove City Council (39, 40). The Brighton project aimed to help develop strategies in self-managed care for older adults, for example, in the context of medication administration, self-monitoring and self-awareness, and self-management of the emotional impact of multiple comorbidities. Therefore, the team focused on a series of interventions for groups of people living in assisted sheltered accommodation to find ways to prevent or delay entry into more intensive and expensive care programs. The lab was developed as a response to address the difficult issue of evidence-based digital health supporting self-managed care and acting as a tool to be used by various stakeholders (41).

**Figure 1:**
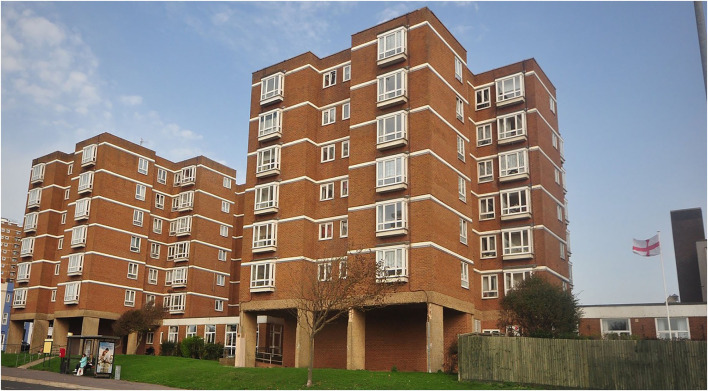
Digital health living lab.

The site chosen to establish the DHLL was a retirement housing scheme recommended by the city council as it meets the requirements in terms of residents' demographics and caring needs. Retirement housing schemes are complexes where senior citizens live independently. They are specifically designed for those over 55, providing easy access, and being efficient and ergonomic. They are maintained by the local council, which provides on-site managers responsible for the safety and day-to-day running of the complex and catering to residents' needs. The site is comprised of 108 flats split into three building blocks, seven stories each, and is linked by a ground floor corridor and communal rooms (Figure 1). Residents were invited to attend a launching event where the DHLL team presented the concept and aims of this initiative and invited them to consider participating by registering their interest and sharing their contact details, consenting to the DHLL contacting them in future, along with basic demographic data including gender and age. Regarding the resident's profile, 81% are 65 years and older, with 58 and 42% female and male subjects, respectively, and 70% with recorded disabilities.

### Step-by-step approach

The literature describes different approaches and methodologies for living labs in other disciplines and sciences (i.e., environmental, green energy, and smart cities). Here, we present our experience and propose an approach directly related to setting up Digital Health Living Lab in the community (42–46).

One of the first decisions is the location that will “host” the living lab. According to our experience, this depends on the focus of the activities in relation to the main stakeholder. Considering that the citizen is always at the center of any activity, the site might be a building block: For example, the sheltered accommodation for our Empowercare project where the building block met the demographic requirements (main stakeholder citizen). If the main stakeholder is the local council, the DHLL can be a neighborhood where the diversity of civic life is more dependent on demographics (i.e., it may be related to environmental factors). It can be based on a community space (or even university dorms) where a group of citizens meet regularly. It can also be at a hospital ward when the main aim is disease orientated.

**Figure 2:**
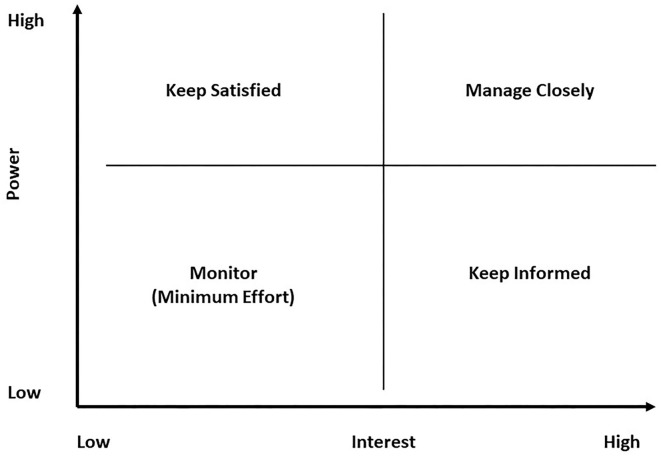
Power/interest matrix of stakeholders.

Once the location is determined, the next step is to create participants' profiles through interviews and understand stakeholder needs through co-production workshops. In Table 1, we present the different stakeholders and their needs from the DHLL, followed by a further Power/interest matrix of stakeholders (Figure 2). Our approach to systematically identify the relevant stakeholders was informed by the study of Manzini (47) and followed the steps for stakeholder selection (48) as described in the AgriLink Living Lab Toolbox (49). Applying these guidelines, we started by defining stakeholders, individuals, and organizations relevant to the living lab residents. The criterion of relevancy was based on the “position” of a citizen within the health and social care system in the UK and was defined as the type of relationship that affected directly or indirectly the residents' lives. As such, we developed a list of stakeholders (Table 1) where the relationship might be direct (i.e., carers, neighbors, and DHLL staff) or indirect (i.e., general health practitioners, local council, and commissioners). The list was also informed by the residents themselves through 1-2-1 interviews exploring who and which individuals and organizations they perceived as affecting their health and wellbeing.

**Table 1:** Scoping DHLL stakeholders' needs.

Citizens of the DHLL • Wellbeing • To live as independently as possible with support as needed • Health and social care that is tailored to them • Choices and the ability to make decisions around health and wellbeing • Seen as an asset, not a burden (what can they do to help!)	Carers/next of kin • Their loved one is being cared for kindly and compassionately • Promote independence but ensure safety and reassurance • What can they do to help?	Staff of DHLL (i.e., site managers of the building) • Happy residents and happy staff • Eager to help but need education and training • Do not want additional responsibilities or workload
General health practitioners •Improved health outcomes • Reduced demand on their services • Do not want additional responsibilities or workload	Secondary care • Improved health outcomes • Reduced demand on their services • Don't want additional responsibilities or workload	Local government implementation plans •Mandate to work on prevention and self-management, better integration of health and social care, and incorporate aspects of local digital roadmap.
Local council/municipality •Improved health outcomes • Reduced demand on services • Needing to improve specific targets (falls/out-of-hours provision/ medication compliance) • Mandate to work on prevention and self-management, better integration of health and social care, and incorporate aspects of local digital roadmaps • Do not want additional responsibilities, workload, or financial commitments • Incorporate digital health as a cornerstone of initiative • Raise their profile through an effective and positive campaign	Commissioners • Improved health outcomes • Reduced demand on services • Needing to improve specific targets (falls/out-of-hours provision/medication compliance) • Mandate to work on prevention and self-management, better integration of health and social care, and incorporate aspects of local digital roadmaps • Incorporate digital health as a cornerstone of initiative • Do not want additional responsibilities, workload, or financial commitments	Academic institution •Mandate to work more collaboratively with civic authorities through the “Leading Places” project • Raise their profile through an effective and positive campaign • Incorporate digital health as a cornerstone of initiative • Research and publications • Ways to incorporate project in the curriculum of health science students
Academic networks •Mandate to work more collaboratively with NHS, academic institutions, and industry • Raise their profile through an effective and positive campaign	Digital innovators • Learning and training through working alongside service users • Innovation • Raise their profile through an effective and positive campaign • Commercialization	Voluntary sector •Keen to be involved and offer their perspective on health and social care • Raise their profile through an effective and positive campaign • May seek or offer funds depending on specific organization and nature of the relationship

Upon the completion of the stakeholder needs identification, we recommend prioritizing these needs reflecting the importance of the stakeholder in relation to the timeframe. In Table 2, we present an example of the prioritization of the needs of the DHLL stakeholders.

**Table 2:** Prioritization of stakeholders' needs.

		**Timing (to address)**	

		**Now**	**Later**
**Activity**	High priority	• To live as independently as possible with support as needed in a safe manner with reassurance • Choice and the power to make decisions around their own health and care needs with plans that are tailored to them • Seen as an asset, not a burden (what can they do to help!) • Their loved one is being cared for kindly and compassionately • Do not want additional responsibilities or workload, or financial commitments	• Improved health outcomes and wellbeing • Reduced demand on their services • Raise their profile through an effective and positive campaign (commonality between multiple stakeholders raises its significance) • Incorporate Digital Health as a cornerstone of initiative • Incorporate project in the curriculum of health science students
	Low priority	• Needing to improve specific targets (falls/out-of-hours provision/ medication compliance) • Keen to be involved and offer their perspective on health and social care • May seek or offer funds depending on specific organization and nature of the relationship	• Mandate to work on prevention and self-management, better integration of healthcare and social care, and incorporate aspects of local digital roadmaps more collaboratively with civic authorities through the “Leading Places” project, academic institutes, and industry • Research and publications • Learning and training through working alongside service users • Innovation • Commercialization

The next steps include regular project management activities, such as time framing (i.e., GANTT chart), risk analysis, and register, followed by the actual implementation of the activities (i.e., testing, evaluation, etc.).

### Projects

**Figure 3:**
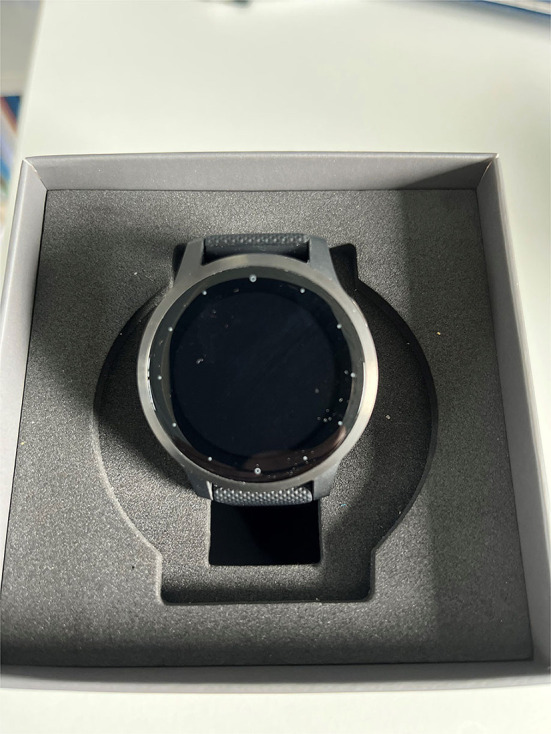
Activity tracker.

**Figure 4:**
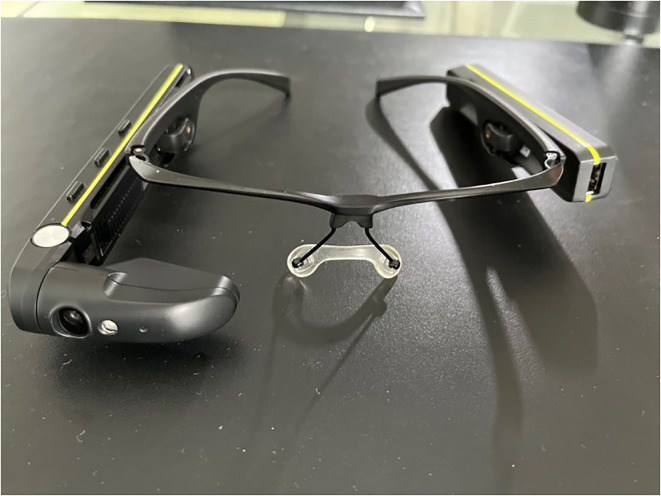
Smart glasses.

Since the establishment of the DHLL, we have utilized it in several cross-disciplinary research projects, with two more notable recent European funded (Table 3). Within these projects, we had the chance to test and evaluate different technologies, including wearables (Figure 3) and smart glasses (Figure 4).

**Table 3:** DHLL projects.

**Project**	**Funding body**	**Aim/content**
EMPOWERCARE (EMPOWERing individuals & communities to manage their own CARE)	Interreg 2 seas	The project aims to create resilient communities and reduce individual frailty and loneliness by developing an approach using research-based solutions and technology to address gaps in the care of the target groups of those aged 65+ and those aged 50+ with at least one chronic condition. The DHLL is utilized as the tool providing a suitable environment to work along with citizens, carers, and healthcare professionals, co-creating, trialing and evaluating digital health solutions that would be able to be implemented across borders and healthcare systems.
AI4HealthSec (A Dynamic and Self-Organized Artificial Swarm Intelligence Solution for Security and Privacy Threats in Healthcare ICT Infrastructures	HORIZON2020	AI4HEALTHSEC proposes a state-of-the-art solution that improves the detection and analysis of cyber-attacks and threats on Healthcare ICT infrastructures and increases the knowledge on the current cyber security and privacy risks within the digital Healthcare ecosystem and among the involved Health operators. As such, it is the first time a DHLL is being utilized as a pilot testing site for the development security framework

## Case study

Sharing our experience working in the DHLL with industrial partners, here we present a case demonstrating how it can contribute to the development of a digital product, in this case, the Kraydel Konnect, throughout different stages of its maturity.

Konnect is an established, easy-to-use home communication system. The system connects the user's TV with a communication hub using the HDMI port on the TV, and this hub connects to the internet *via* the User's home Wi-Fi network or mobile 4G signals. The hub enables video calls by connecting with standard video-calling platforms (i.e., Vonage and Zoom) for TV-based video calling.

Konnect's user interface is designed as a carousel system (i.e., the user cycles through options on their TV) which the user navigates through by simply responding with a “yes” or “no” from the specially designed remote control. Kraydel has several user-related features, such as the ability to respond to questionnaires and surveys through the TV, which can be used to gather user-related insights such as wellbeing assessments. Callers can use the Konnect app on their smartphones (available for iOS and Android) to make video calls to the user's TV. The system also offers so-called sofa-to-sofa communication. For example, any individual (relative, carer, and healthcare professional) who has a Konnect unit can make calls to the user's TV from their own Konnect unit as long as the user allows access in advance. Users can also upload photos from their phones and upload videos through a dashboard that can be viewed on the TV.

**Figure 5:**
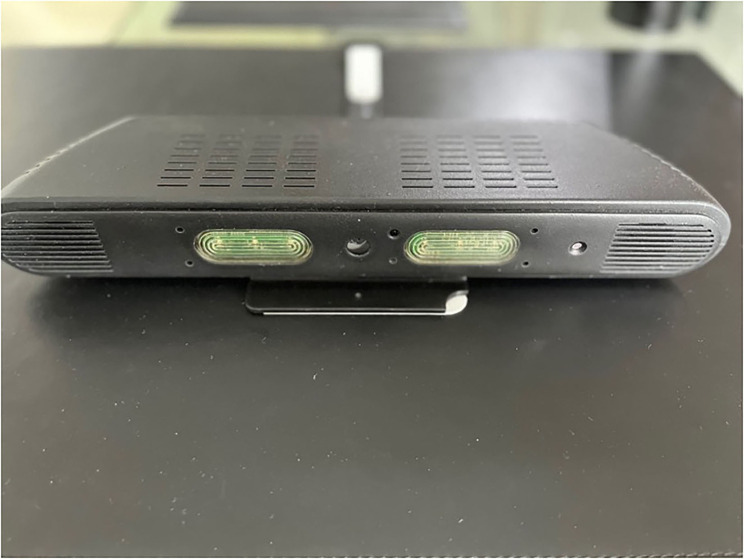
Kraydel Konnect first prototype.

**Figure 6:**
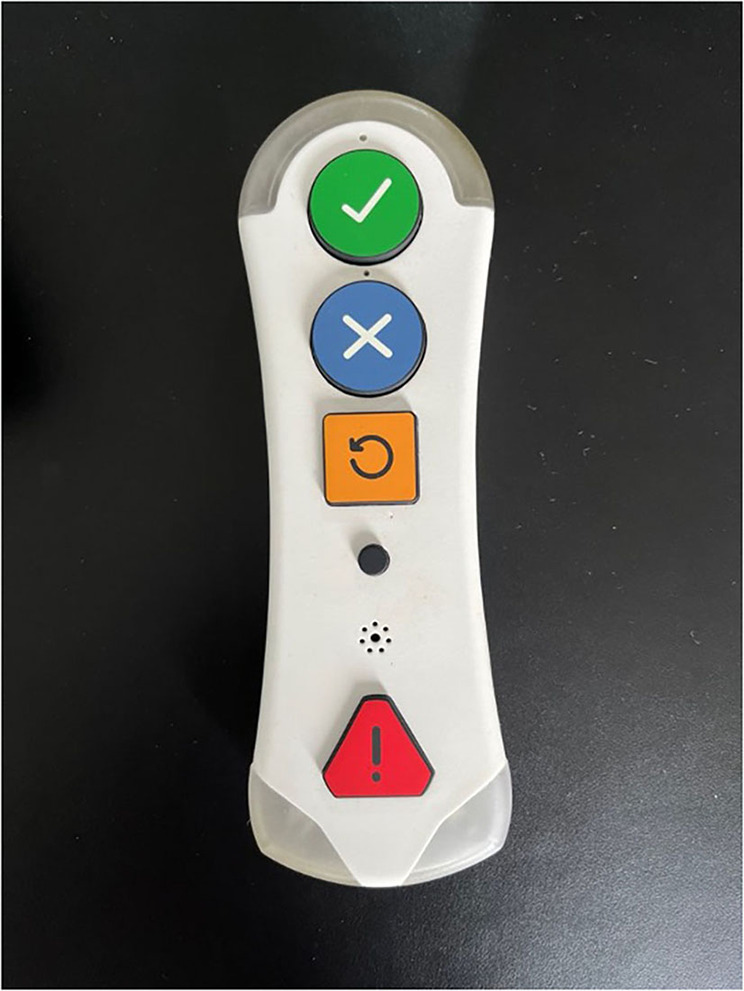
Kraydel Konnect remote control first prototype.

Kraydel's aim was to work with residents of the DHLL and through co-production workshops to share the first prototypes developed in their labs and receive end user's input toward the further development and finalization of these early versions of their devices. As such initial prototypes (Figures 5, 6) of the Konnect units have been used by residents in the DHLL, providing valuable input and feedback for further adjustments.

**Figure 7:**
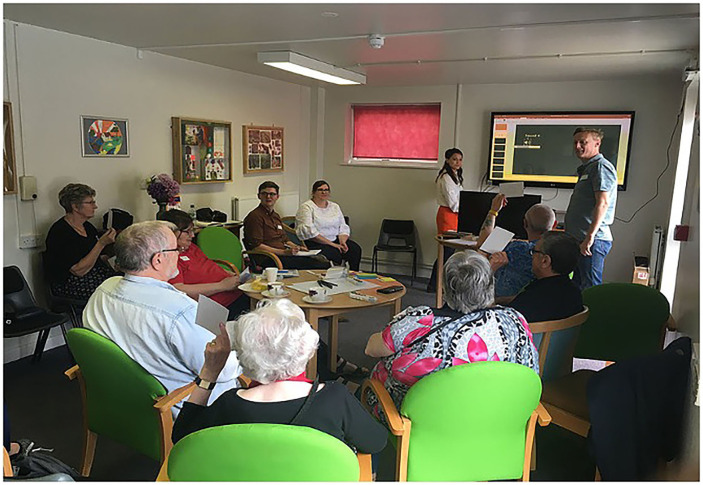
Co-creation workshop.

**Figure 8:**
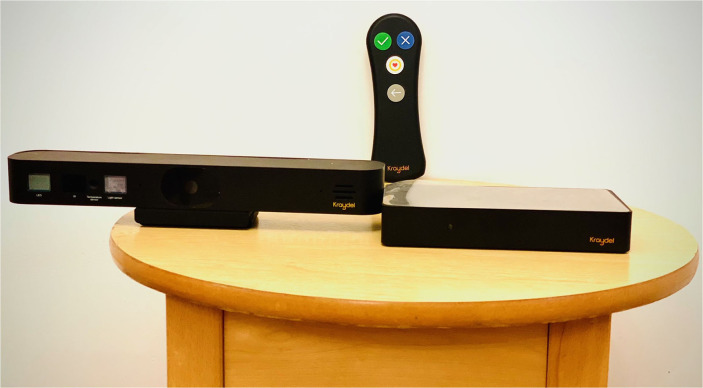
Kraydel Konnect updated version.

The testing and development continued with further co-creation workshops, focus groups, and individual interviews (47–49) (Figure 7), ensuring the updated hardware design and the user interface is easy and straightforward and do not provoke technophobia (Figure 8). The co-creation workshops took the shape of 1-2-1 sessions between residents and the developers' devices, supported by user experience and design thinking professionals, exploring technical aspects (shape, materials, colors, usage, and dexterity). At the same time, the focus groups were utilized to gather qualitative feedback on usability feasibility and applicability from both the residents and additional stakeholders (in this case, DHLL staff, residents' carers, and friends). In total, the project included two 1-2-1 sessions as described earlier and six focus groups.

**Figure 9:**
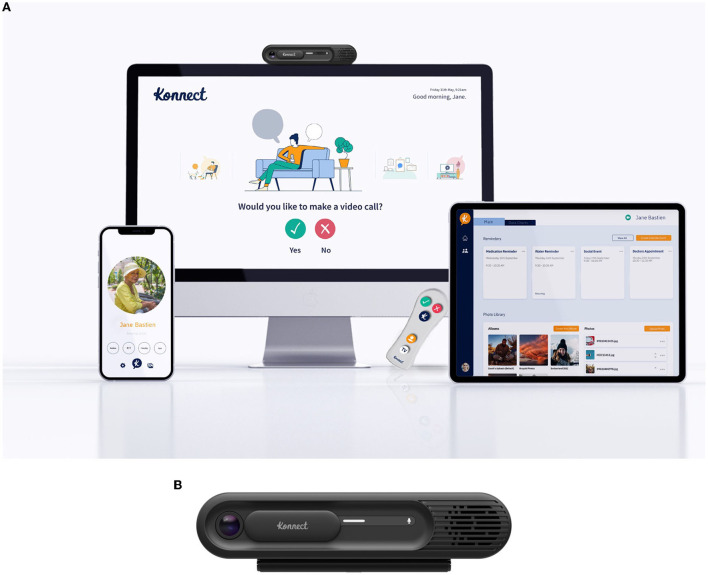
**(A, B)** Kraydel Konnect current version.

The current Konnect version is also a wellbeing monitoring system that uses onboard sensors (for room temperature and physical movement in the TV room) and has Bluetooth capability with a wide range of third-party devices that are in the process of being integrated. Remote secure cloud for storage and processing of data and Application Programming Interfaces (APIs) connecting local devices to Konnect are already established for digital thermometers and pulse oximeters, and heart rate and to feed data to service provider personnel for remote assessment (Figures 9A, B).

As discussed earlier, existing literature (31) highlights the benefits of various types of living labs, like our DHLL.

### Lessons learned

In this case study, we share lessons learned from this development as well as some unique advantages linked to such a community-based DHLL:

- Real-life environment. During our testing and evaluation activities, it became apparent that conducting these in participants' own homes provided a more original experience as reflected by their feedback.
- Costs. As discussed in the introduction, establishing an LL, for example, in a university environment or a municipality building, is accompanied by costs related to the actual room, facilities, maintenance, utility bills, and staff. On the contrary, our experience showed that establishing a DHLL in the community has the advantage of lower costs. It can be set up in an existing site without any additional costs for extra physical spaces and their accompanying expenses, such as utility bills and/or maintenance as these are covered by the local council. In our case, the used spaces for any of the scheduled activities are either the communal spaces or smaller rooms where we conduct interviews or focus groups.
- Sustainability. Further to the maintenance of the physical spaces of living labs, their sustainability requires the investment of personnel and time for traveling and transport, bringing participants together and keeping them engaged. In our case, an important advantage is a fact that the community-based DHLL is “self-sustained.” The group of participants engages in the labs' activities in their own familiar spaces (homes and communal areas) without the need to travel to the university or to another site to continue networking with fellow residents even when there are no active projects. As a result, there is no need for continuous presence or visits of academic staff. In addition, the presence of on-site managers provides the advantage of fast recruitment and resuming of activities once a new project starts through the dissemination of any required material and invitations.
- A benefit for any SMEs utilizing such a DHLL is their opportunity to showcase their solutions directly to end users but also stakeholders, including commissioners and decision-makers.

### Limitations

Setting and maintaining a living lab can be accompanied by many limitations, but in this case study, we have an opportunity to share our experience reflecting on a single development outside of having explored a summative project evaluation. Based on this experience, the team identified certain limitations that, if considered going forward, would expand the DHLL benefits.

One of the limitations is the demographics of the residents, where, although there is a diverse group of citizens living on this site, these might not represent the diverse community beyond the living lab. A way to mitigate this limitation would be by including more sites from areas across the council that would include hard-to-reach and/or vulnerable populations (i.e., minorities and learning disabilities).

Another limitation of this single development comes from the voluntary commitment of the residents. This results in working with residents that might already be tech-savvy and eager to contribute to such testing. This may exclude valuable input from no digitally literate citizens that hesitate to volunteer in such testing. A future solution could be to increase the number of living labs and provide incentives to potential participants.

## Conclusion

In this single-case study, the DHLL proved to be an open innovation ecosystem as it brought together multiple stakeholders sourcing ideas for a small business enterprise, contributing effectively to the user-centered development of the described digital health solution. Following the approach shared in our article, we believe that establishing DHLLs in the community and engaging with the right stakeholders can be a streamlined and straightforward process with the subsequent benefits described.

## Data availability statement

The original contributions presented in the study are included in the article/supplementary material, further inquiries can be directed to the corresponding author.

## Ethics statement

Written informed consent was obtained from the individual(s) for the publication of any potentially identifiable images or data included in this article.

## Author contributions

All authors listed have made a substantial, direct, and intellectual contribution to the work and approved it for publication.
